# Bed Curtains

**Published:** 1856-04

**Authors:** 


Bed Curtains are unhealthy, because they confine the air around us while we sleep; a canary bird will die in a night, susped in that situation.

